# Supplementary material for: The impacts of economic globalization on agricultural value added in developing countries
Source: PLoS One. 2021 Nov 17;16(11):e0260043. doi: 10.1371/journal.pone.0260043 (PMC8598014; doi:10.1371/journal.pone.0260043)
Supplement: Attachment — Submitted filename: Review Letter.docx [file pone.0260043.s002.docx]

**The Impacts of Economic Globalization on Agricultural Value Added in Developing
Countries**

I find the paper to be very interesting. **Major revision**

**Comments#1**: Abstract

1) A brief statement of motivation of the study is required.
2) A brief statement of contribution of the study is required.
3) Discussion of the results should focus on the main objective, Policy part is missing.
4) The abstract should indicate clearly the objectives, method ology, findings and possibly recommendation.

**Comments#2**: Introduction

1) Explain the research problem regarding the main context related to EG and agriculture value added in the context of developing economies

2) Why is the problem very important and why focus on these economies?

3) State the core purpose of the study and how authors intend to achieve it methodologically and data wise.

4) How different is the current study from previous attempts and how significant is this contribution.

5) Extend the contribution of the study.

**Comments#3**: If you do a section for theoretical connection, it would be more interesting and improved. If possible, do it.

**Comments#4** there is no numbering. please insert numbers like 1. Introduction, 2. Review of Literature etc

**Comments#5:** what is the reason to these set of variable justify.

Comments#6: Add more policies based on your finding with linking literature.
