# Supplementary material for: The impacts of economic globalization on agricultural value added in developing countries
Source: PLoS One. 2021 Nov 17;16(11):e0260043. doi: 10.1371/journal.pone.0260043 (PMC8598014; doi:10.1371/journal.pone.0260043)
Supplement: Attachment — Submitted filename: Response to Reviewers.docx [file pone.0260043.s003.docx]

**Response to the reviewer comments**

| **Number** | **Reviewer comment** | **Response** |
| --- | --- | --- |
| 1 | Introduction section should clearly convey the aim of the paper, which is missing | We add it in the line 150 |
| 2 | the explanation of all types of panel estimation is not required, rather the author shall suggest why they have used the mentioned technique and not other techniques. | 1. We are hoping to be able to show the steps of panel data regression analysis in order to explain the manuscript's analytical method 2. We have explained the reasons for using this analytical method in the methodology section (line 311-315) |
| 3 | It is suggested to use robustness test in the study | Dear reviewer, I have tested the robustness of this research, including multicollinearity, heteroscedasticity and autocorrelation (Table 2), therefore I believe I have met your request. |
| Abstract | | |
| 1 | A brief statement of motivation of the study is required | I add it in the line 14-16 |
| 2 | A brief statement of contribution of the study is required | I add it in the line 16-19 |
| 3 | Discussion of the results should focus on the main objective; Policy part is missing | The results already focus on the main objective and I add policy in the line 26-29 |
| 4 | The abstract should indicate clearly the objectives, methodology, findings and possibly recommendation | Done |
| Introduction | | |
| 1 | Explain the research problem regarding the main context related to EG and agriculture value added in the context of developing economies | I add it in the line 67-85 and 121-135 |
| 2 | Why is the problem very important and why focus on these economies? | I add it in the line 68-101 |
| 3 | State the core purpose of the study and how authors intend to achieve it methodologically and data wise | I add the study aim at line 150-152. Meanwhile, we explain how to achieve it in the method section |
| 4 | How different is the current study from previous attempts and how significant is this contribution | In the introduction, we presented various studies on the impact of EG on agriculture but so far there has been no research on its impact on AVA. We also add the contribution of this research for both theory and practice on lines 142-148 |
| 5 | Extend the contribution of the study | We add it in the line 142-148 |
|  | If you do a section for theoretical connection, it would be more interesting and improved. If possible, do it. | We add it in section 2 |
|  | there is no numbering. please insert numbers like 1. Introduction, 2. Review of Literature etc | We add numbering in every section |
|  | what is the reason to these set of variables justify | We explain it in introduction section (line 137-142) and literature section |
|  | Add more policies based on your finding with linking literature | We add it in the section 7 |
|  | You can also consider the following paper to enhance implications:  Gozgor, G. (2019). Effects of the agricultural commodity and the food price volatility on economic integration: an empirical assessment. Empirical Economics, 56(1), 173-202. | We add it as reference |
